# Dual-tissue transplantation versus osteochondral autograft transplantation in the treatment of osteochondral defects: a porcine model study

**DOI:** 10.1186/s13018-023-03964-6

**Published:** 2023-07-05

**Authors:** Rongmao Shi, Gang Wang, Zhian Chen, Libo Yuan, Tianhua Zhou, Hongbo Tan

**Affiliations:** 1 Department of Orthopaedic Surgery, 920th Hospital of Joint Logistics Support Force, Kunming, Yunnan China; 2 Department of Orthopaedic Surgery, Xiangzhou Distract People’s Hospital, Xiangyang City, Hubei Province China

**Keywords:** Osteochondral injury, Articular cartilage, Animal model, Bone graft, Osteochondral autograft transplantation, Autologous cartilage chips

## Abstract

**Background:**

Osteochondral injury is a common sports injury, and hyaline cartilage does not regenerate spontaneously when injured. However, there is currently no gold standard for treating osteochondral defects. Osteochondral autograft transplantation (OAT) is widely used in clinical practice and is best used to treat small osteochondral lesions in the knee that are < 2 cm^2^ in size. Autologous dual-tissue transplantation (ADTT) is a promising method with wider indications for osteochondral injuries; however, ADTT has not been evaluated in many studies. This study aimed to compare the radiographic and histological results of ADTT and OAT for treating osteochondral defects in a porcine model.

**Methods:**

Osteochondral defects were made in the bilateral medial condyles of the knees of 12 Dian-nan small-ear pigs. The 24 knees were divided into the ADTT group (*n* = 8), OAT group (*n* = 8), and empty control group (*n* = 8). At 2 and 4 months postoperatively, the knees underwent gross evaluation based on the International Cartilage Repair Society (ICRS) score, radiographic assessment based on CT findings and the magnetic resonance observation of cartilage repair tissue (MOCART) score, and histological evaluation based on the O'Driscoll histological score of the repair tissue.

**Results:**

At 2 months postoperatively, the ICRS score, CT evaluation, MOCART score, and O'Driscoll histological score were significantly better in the OAT group than the ADTT group (all *P* < 0.05). At 4 months postoperatively, the ICRS score, CT evaluation, MOCART score, and O'Driscoll histological score tended to be better in the OAT group than the ADTT group, but these differences did not reach statistical significance (all *P* > 0.05).

**Conclusions:**

In a porcine model, ADTT and OAT are both effective treatments for osteochondral defects in weight bearing areas. ADTT may be useful as an alternative procedure to OAT for treating osteochondral defects.

**Supplementary Information:**

The online version contains supplementary material available at 10.1186/s13018-023-03964-6.

## Background

Osteochondral injury involves both the articular cartilage and the subchondral bone. Such osteochondral injuries are common sports injuries as a result of trauma or secondary to an osteochondritis dissecans (OCD) lesion. Previous studies have estimated that the prevalence of OCD in the adolescent population is 15–30 per 100,000 adolescents [1, 2]. Furthermore, approximately 10% of knee sports injuries in adolescent patients are accompanied by cartilage lesions [3]. Symptoms of focal cartilage or osteochondral defects include pain, swelling, and stiffness, which limit the ability of the patient to perform activities.

Articular cartilage is hyaline cartilage that consists of a dense extracellular matrix and embedded chondrocytes, without any blood vessels, lymphatic vessels, or nerves. Hyaline cartilage lacks a blood supply and is instead supplied synovial fluid, resulting in the poor self-repairing ability of cartilage defects. Focal cartilage injury may result in cartilage loss in the same subregion [4], increase the risk of incident cartilage damage in the same tibiofemoral joint compartment regardless of the defect depth [5], and finally lead to osteoarthritis; at least 12% (and possibly upwards of 30–40%) of osteoarthritic cases are believed to be of posttraumatic origin [6, 7].

The current options for cartilage defects include osteochondral autograft transplantation (OAT) [8–11], marrow-stimulation techniques (e.g., microfracture) [12–14], autologous chondrocyte implantation (ACI) [15, 16], osteochondral allograft transplantation [17–20], particulated juvenile allograft cartilage [21, 22], matrix-induced ACI (MACI) [23, 24], autologous cartilage chips (ACC) [25–27], and osteochondral tissue engineering [28]. These various treatment methods each have associated problems such as inconsistent clinical and biological outcomes, the need for a two-stage procedure, high costs, limited indications, limited donor material, donor site morbidity, and graft rejection.

OAT or mosaicplasty addresses osteochondral lesions while maintaining the hyaline cartilage by replacing the defect with an osteochondral autograft [29]. OAT is widely used in clinical practice and is a best used to treat small osteochondral lesions in the knee that are < 2 cm^2^ in size [30]. The advantages of OAT are that it is a single-stage procedure, has a lower cost compared with allograft transplantation, and has the ability to treat lesions with subchondral bone involvement. Furthermore, using the patient’s native cartilage and living bone should theoretically improve the healing potential. However, a limitation of OAT is that it can be difficult to contour match the donor cartilage to the lesion to create a congruent surface [30]. In addition, larger lesions are more difficult to treat with OAT because they necessitate a mosaic construct and may result in donor site morbidity [30].

In 2015, a new technique called autologous dual-tissue transplantation (ADTT) was introduced to clinically treat osteochondral defects [31]. Treatment of OCD with ADTT results in very good subchondral bone restoration and good cartilage repair, with significant improvements in patient-reported outcomes at 1 year postoperatively [31]. Hence, ADTT is thought to be a promising, low-cost treatment option for osteochondral injuries. ADTT is a combination of fragmented autologous bone press-fitted into the defect bed and autologous cartilage chips embedded in fibrin glue to cover the bone graft [31]. ADTT has a broader indication and more donor cartilage than OAT and has no donor site morbidity; however, there are few histological and clinical studies of ADTT. Therefore, the present study aimed to compare the histological and radiographic results of ADTT and OAT in a porcine model of osteochondral defects. The hypothesis was that ADTT would achieve similar histological and radiographic results to OAT in the treatment of osteochondral defects.

## Methods

### Animal preparation

Twelve skeletally mature Dian-nan small-ear pigs of either sex without osteoarthritic injury or degeneration were used in this study. The pigs had an average weight of 37.20 ± 2.43 kg (range 32.56–39.78 kg) and an average age of 19.38 ± 1.06 months (range 18.0–21.5 months). All pigs were purchased from the Small Pig Animal Experiment Center of Yunnan Agricultural University and were kept in accordance with the “Standard of Care for Laboratory Animals.” This study was approved by the Ethics Committee of the 920th Hospital of Joint Logistics Support Force (No. 920IEC/AF/61/2021-01.1).

### Study design

The left knees of eight pigs comprised the OAT group (*n* = 8), while the right knees comprised the ADTT group (*n* = 8). The remaining four pigs (eight knees) were used as the empty control group (*n* = 8). An osteochondral defect (8 mm diameter, 8 mm depth) was created in each medial femoral condyle. Six pigs (two from each group) were euthanized at 2 and 4 months postoperatively, and knee samples were removed for gross observation of the repair tissue of the defects, computed tomography (CT) and magnetic resonance imaging (MRI) for imaging evaluation, and histological staining for histological evaluation.

### Surgical procedures

The pigs were premedicated with intramuscular injections of xylazine (0.1 ml/kg; Shandong Luwei Co., Ltd., China) and atropine (1.5 ml/kg; Shandong Huamu Pharmaceutical Co., Ltd., China) to induce muscle relaxation and were then intravenously injected with pentobarbital sodium (3%, 1 ml/kg; Shandong Huamu Pharmaceutical Co., Ltd, China) to induce anesthesia. Once the anesthetic effect was stable, the operation was started. All surgeries were performed by the same senior joint surgeon (Dr. Hongbo Tan, Chief Physician) at the Animal Experimentation Center of the 920th Hospital of Joint Logistics Support Force. Each knee was prepared by removing the hair and performing routine disinfection. Ropivacaine (10 ml, 100 mg, Hengrui Pharmaceutical Co., Ltd., China) was administered to the skin and periarticular tissue. A 4–5-cm longitudinal skin incision was made over the middle of the anterior knee joint. Access to the joint was achieved via an incision along the medial border of the quadriceps tendon through the medial edge of the patella to the medial aspect of the tibial tuberosity. The patella was dislocated laterally to expose the weight-bearing area of the medial femoral condyle. A hand drill was used to create a critical-size drill hole (8 mm diameter, 8 mm depth) as an osteochondral defect in the medial femoral condyle. The defect was cleared with a scraping spoon, and the removed cancellous bone was set aside. The hole and knee joint cavity were then irrigated with sterile saline.

For the knees of the ADTT group, the cylindrical osteochondral block from the drill hole was separated into cartilage and cancellous bone. A drill hole was made in the proximal tibial to harvest a biopsy of autologous cancellous bone; this biopsy material was added to the cancellous bone obtained from the femoral drill hole and was broken into fragments. These fragments were press-fitted into the bony part of the drill hole in the medial femoral condyle to a level flush with the surface of the subchondral bone. A sharp knife was used to harvest articular cartilage from non-weightbearing areas (femoral intercondylar fossa, medial edge of the medial femoral condyle, and medial and lateral edges of the trochlea). The harvested cartilage was then placed in a metal basin, and a scalpel was used to chip the cartilage into pieces of approximately 0.5–1 mm^3^. The autologous cartilage chips were embedded in a Porcine Fibrin Sealant Kit (Guangzhou Beixiu Biotechnology Co., China) and placed onto the bone graft in alignment with the adjacent cartilage surface. The fibrin glue was allowed to set for 5 min. The patella was then replaced, and the stability of the graft was tested with full-range motions.

For the knees of the OAT group, OAT was performed as described previously [9]. A cylindrical osteochondral graft (8 mm diameter, 8 mm depth) was harvested from the lateral trochlear flare superior to the sulcus terminalis. A graft pusher was used to advance the osteochondral plug into the osteochondral defect until it was flush with the surrounding native cartilage. Finally, a tamp was used to gently compress the graft until it was flush. The donor site was filled with subchondral cancellous bone taken from the recipient area.

For the knees of the empty control group, no treatment was performed after the preparation of the osteochondral defect in the medial femoral condyle.

The knee joint cavity was irrigated with sterile saline, and the subcutaneous tissue and skin were sutured using absorbable sutures. The pigs were returned to the animal center and allowed to bear weight and move freely without any activity restriction. Each pig was injected intramuscularly with sodium penicillin (20,000–30,000 units/kg, Inner Mongolia Federal Animal Protection Drug Co., Ltd., China) twice daily for 1 week to prevent postoperative infection.

### Gross evaluation

Pigs were euthanized by intravascular injection of pentobarbital (0.4 ml/kg, Shandong Xinhua pharmaceutical Co., Ltd., China) at 2 or 4 months postoperatively. All knee samples were collected. Gross evaluation of the cartilage surface of the repair tissue was assessed using the International Cartilage Repair Society (ICRS) scoring system (Additional file 1: Table S1) [32]. Three joint surgeons who were blinded to the treatment methods scored all specimens individually, and the mean values were used in analysis.

### Radiological evaluation

After euthanasia, the pigs were immediately sent to the 920th Hospital of Joint Logistics Support Force for CT (United Imaging Healthcare UCT 510, China) and MRI (United Imaging Healthcare UMR 560, China) examinations. The repair tissue was quantitatively assessed using the magnetic resonance observation of cartilage repair tissue (MOCART) scoring system, a widely accepted scoring system based on MRI [33, 34]. We used a percentage-based MOCART scoring system presented by Quirbach et al. (Additional file 1: Table S2) [35]. Three senior radiologists who were blinded to the treatment groups scored the knees individually, and the mean values were analyzed.

### Histological evaluation

Target portions were separated from freshly collected samples and fixed with 4% polyformaldehyde for 48 h. After the decalcification of the sample was completed, it was dehydrated through a graded alcohol series, made transparent with xylene, soaked in wax, and then embedded. Specimens were cut along the sagittal plane of the center of the defect area into 10-µm-thick sections using a hard tissue slicer and placed in an oven at 60 °C overnight. The sections were deplasticized in dimethoxyethyl acetate and rehydrated before staining. All samples were stained with hematoxylin and eosin, safranin-O, and toluidine blue. Histological evaluation was performed using the O'Driscoll histological score (Additional file 1: Table S3) [36] by three pathologists who were not involved in the experiment. The three pathologists each scored the results, and the mean values were used in the analysis.

### Statistical analysis

All results are expressed as mean ± standard deviation. As this was an experimental animal study with a relatively small sample size, the Shapiro–Wilk test was used to test the normality of the sample data. Statistical analysis was performed using SPSS 22.0 software (SPSS Inc., Chicago, Illinois, USA). *P* values of < 0.05 were taken to indicate statistical significance.

## Results

All surgeries were successful, and all postoperative incisions were healed and free of infection with no complications.

### Gross evaluation

**Fig. 1:**
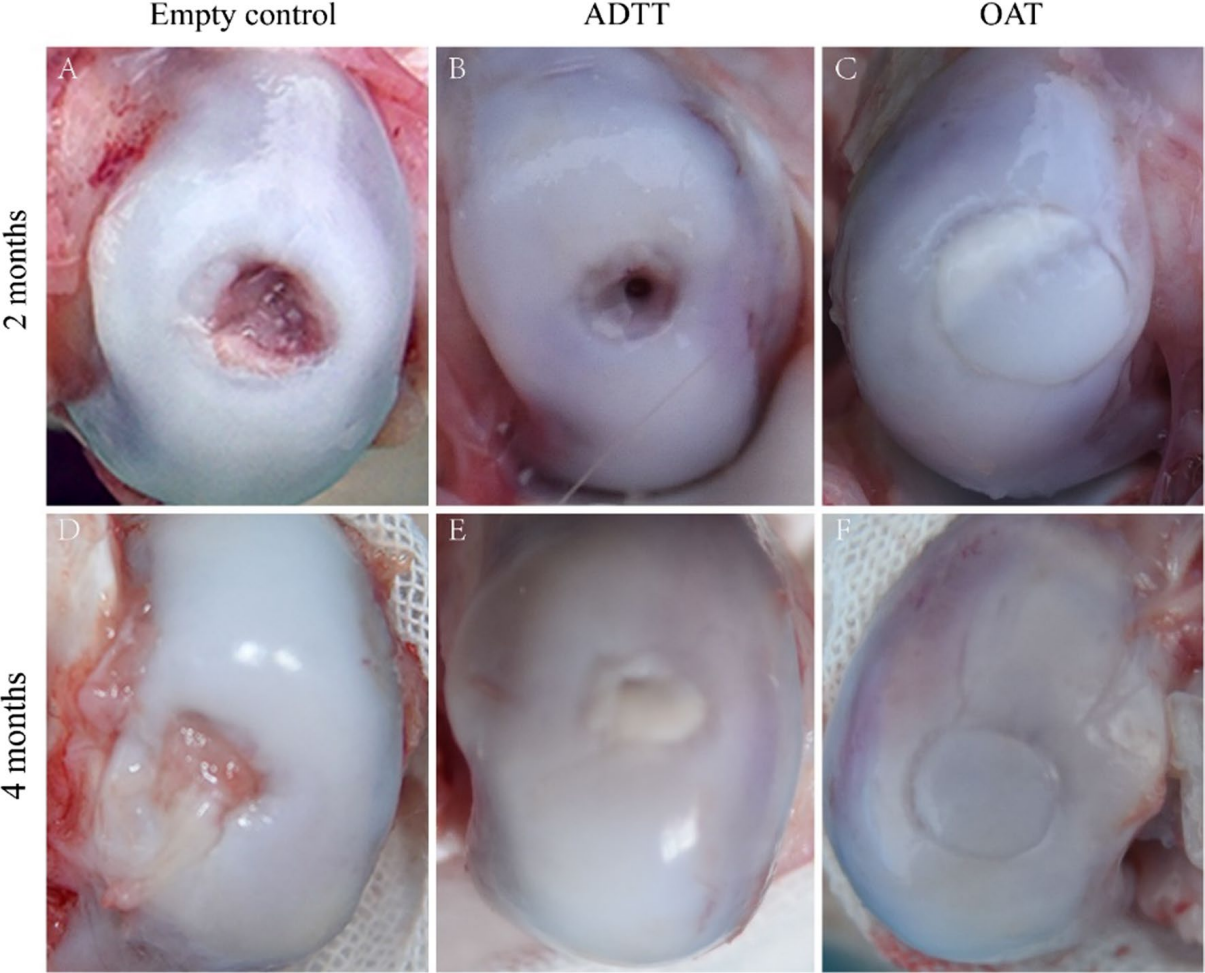
Representative macroscopic images of the empty control, ADTT, and OAT groups at 2 and 4 months postoperatively

The surfaces of the osteochondral defects were better in both the ADTT and OAT groups than the empty control group and had no hyperemia, edema, or inflammatory response at 2 and 4 months postoperatively. In all knees in the empty control group, the osteochondral defects had failed to heal by filling fibrous tissue healing at 2 and 4 months postoperatively. In the ADTT group at 2 months postoperatively, one osteochondral defect was classified as ICRS grade IV because it showed severely abnormal healing with a 2-mm-wide, 2-mm-deep, circular cartilage depression at the center of the defect (Fig. 1B). The surface cartilage of the grafts in the OAT group at 2 months postoperatively was good except for one knee that had graft cartilage protruding approximately 1 mm from the surrounding articular surface (Fig. 1C) and one knee that had a slight depression in the surface cartilage at 4 months postoperatively (Fig. 1F). The ICRS scores are shown in Table 1.

**Table 1:** ICRS, MOCART, and O’Driscoll histological scores

Score	Group	2 months	4 months	*t* value	*P* value
ICRS score					
	ADTT	4.50 ± 1.29	6.25 ± 2.22	0.81	0.48
	OAT	10.25 ± 0.96	9.25 ± 3.10	0.78	0.50
	Empty control	1.00 ± 0.82	2.25 ± 0.96	1.46	0.24
	*t* value of ADTT-OAT	23.00	1.68		
	*P* value of ADTT-OAT	0.00	0.19		
MOCART score					
	ADTT	50.00 ± 12.25	53.75 ± 18.43	0.33	0.76
	OAT	80.00 ± 7.07	75.00 ± 4.08	0.93	0.42
	Empty control	16.25 ± 7.5	20.00 ± 4.08	0.92	0.43
	*t* value of ADTT-OAT	3.80	2.03		
	*P* value of ADTT-OAT	0.03	0.14		
O’Driscoll score					
	ADTT	11.25 ± 1.5	16.75 ± 1.71	4.62	0.02
	OAT	16.00 ± 1.63	20.00 ± 1.63	2.83	0.07
	Empty control	6.25 ± 2.06	10.75 ± 1.08	3.12	0.05
	*t* value of ADTT-OAT	9.92	3.15		
	*P* value of ADTT-OAT	0.00	0.05		

### Radiological results

**Fig. 2:**
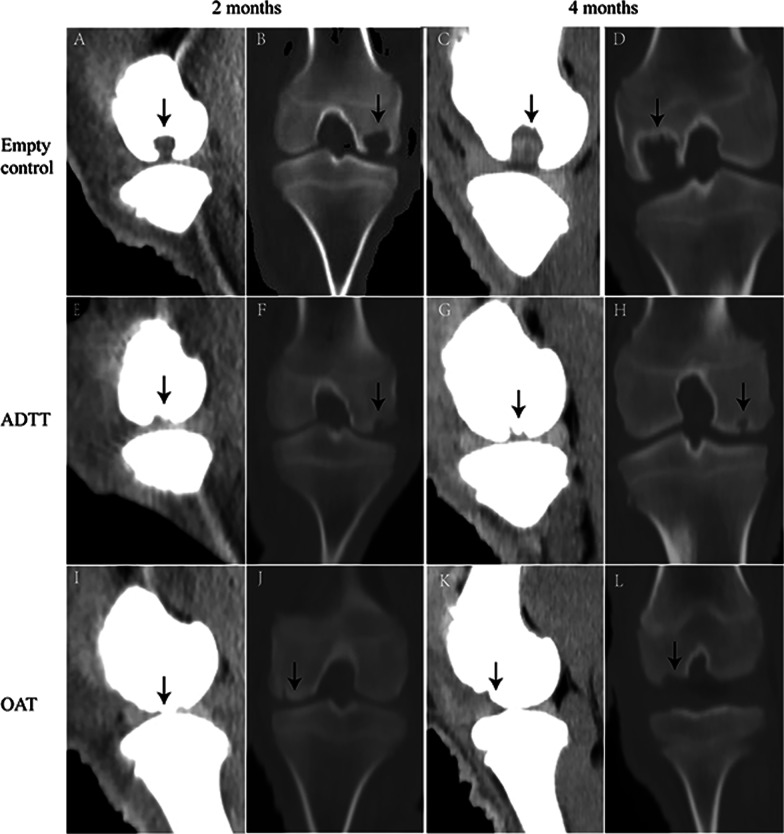
Representative sagittal and coronal CT images of the empty control, ADTT, and OAT groups at 2 and 4 months postoperatively

**Fig. 3:**
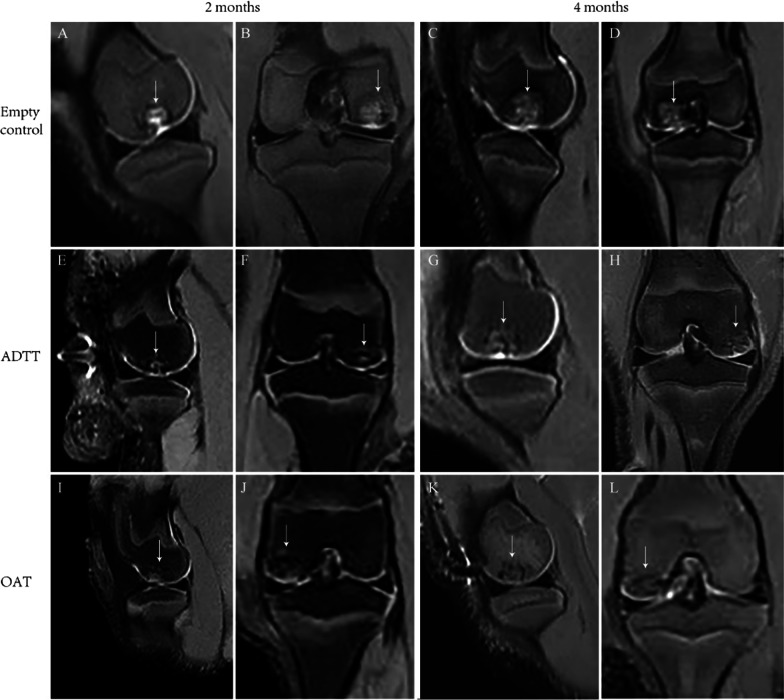
Representative sagittal and coronal magnetic resonance images of the empty control, ADTT, and OAT groups at 2 and 4 months postoperatively

The CT results are shown in Fig. 2. In the empty control group, there were no visible repair changes in the defect at 2 and 4 months postoperatively. Healing of the grafted cancellous bone with surrounding cancellous bone was better in the ADTT group than in the OAT group. There was a clear demarcation between the transplanted and adjacent cancellous bone in the OAT group (Fig. 2J). The surface of the transplanted cancellous bone was uneven in the ADTT group. The MRI results are shown in Fig. 3, and the MOCART scores are shown in Table 1.

### Histological evaluation

**Fig. 4:**
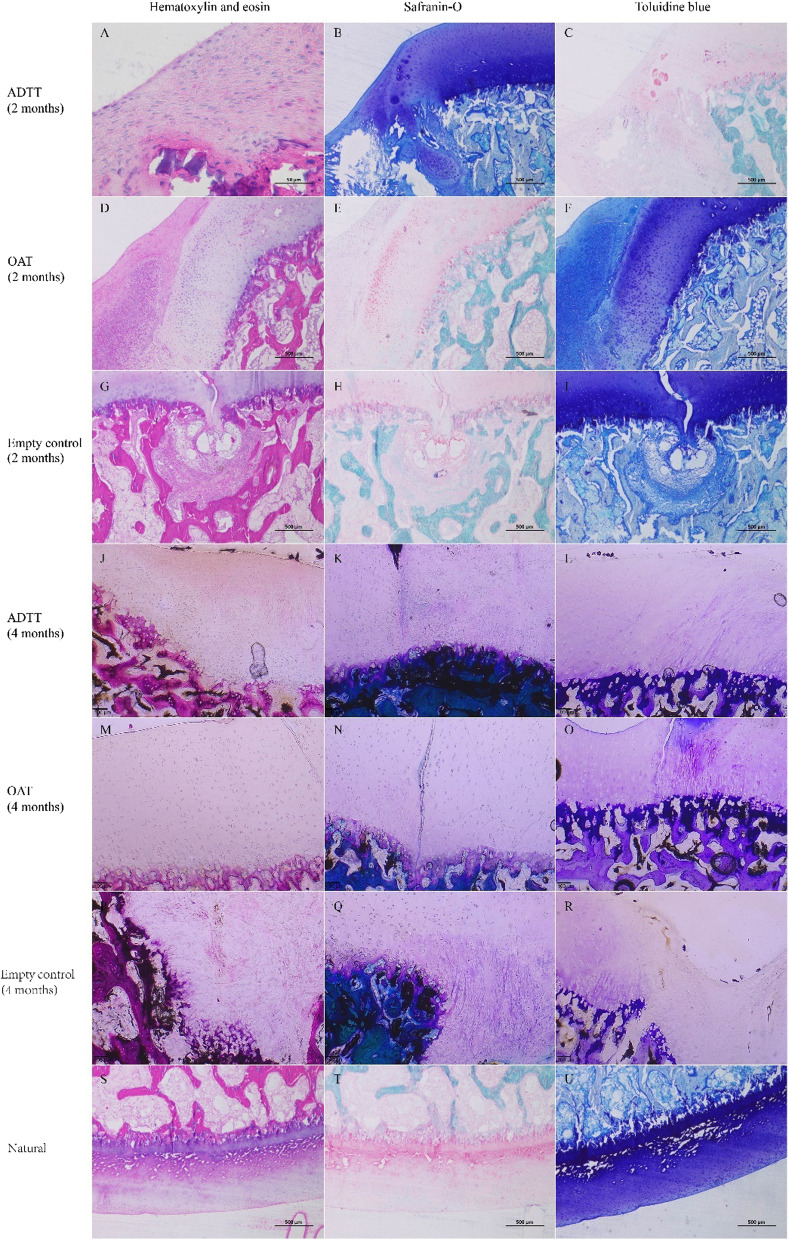
Histological results of the empty control, ADTT, and OAT groups at 2 and 4 months postoperatively. Slides are stained with hematoxylin and eosin, safranin-O, and toluidine blue

The histological results are presented in Table 2 and Fig. 4. The empty control group showed the worst repair results. At 4 months postoperatively, the defects in both the ADTT and OAT groups had good filling and a repaired cartilage layer with nearly normal thickness. The O'Driscoll histological scores are shown in Table 1.

**Table 2:** Histological results of the empty control, ADTT, and OAT groups based on hematoxylin and eosin, safranin-O, and toluidine blue staining

Treatment group	Time-point (months)	Cartilage layer	Cellular morphology	Transplant boundary
ADTT	2	Thin, subchondral tidemark was unclear and discontinuous	Small amount of rounded, irregularly chondrocyte, unevenly distributed	Unclear
	4	Close to normal thickness, tidemark was clear and continuous	More hyaline chondrocytes, small number of fibroblasts, nearly normal cellular morphology	Unclear
OAT	2	Thin, failed to be flush with the normal, tidemark was clear and continuous	Uniform arrangement and distribution of chondrocytes	Clear
	4	Normal thickness, tidemark was clear and continuous	Uniform arrangement and distribution of chondrocytes	Clear
Empty control	2	No cartilage layer	Small number of fibrocartilages	No graft
	4	No cartilage layer	Small number of fibrocartilages	No graft

## Discussion

The primary finding of the present study was that ADTT achieved comparable results to OAT in terms of the gross, radiological, and histological outcomes in the treatment of osteochondral defects in the knees of Dian-nan small-ear pigs. Furthermore, we confirmed that OAT achieved good radiological and histological results in the early postoperative period, while the autologous cartilage chips took several months to repair the osteochondral defects in the ADTT group. Finally, our study showed that the Dian-nan small-ear pig is reliable as a model of osteochondral defects.

OAT with mosaicplasty was first used to treat knee cartilage damage in 1992 and was reported in 1994 by Hangody et al. [37]. OAT is now widely used to treat osteochondral defects. The best indication for OAT is small (< 2 cm^2^) cartilage defects or osteochondral defects [29, 30]. Theoretically, using the patient's native cartilage and living bone to treat osteochondral defects leads to optimal healing and clinical outcomes. Current evidence shows improved clinical results with OAT for osteochondral defects treated when compared with preoperative conditions. Patients can reportedly return to sports as early as 6 months after OAT. A long-term (10–25 years) study showed that knee mosaicplasty achieves good long-term results in soccer players, with no significant decrease in functional scores compared with other studies even at 20 years postoperatively [8]. Similarly, a retrospective multicenter survey showed good clinical outcomes at a mean follow-up of 8.7 years (range 5–20 years) after mosaicplasty for talar cartilage repair, with no deterioration in functional results during long-term follow-up [38]. OAT has also been investigated in experimental studies using animal models [39–42]. However, as these animal studies did not use CT and MRI to evaluate the repair outcomes, we could not compare the results with the radiological results. Clinical studies have evaluated the repair outcome of OAT using the MOCART score.

Clinical studies have evaluated the repair outcome of OAT using the MOCART score. Keszég et al. reported the long-term (10–25 years) outcomes of knee osteochondral autologous transplantation in 73 soccer players, among 25 patients who underwent MRI at the last follow-up; the mean MOCART score was 70.8 ± 18.12 (25–95) [8]. As the MOCART scores are based on MRI, we think that the results would be comparable between animals and humans. In the present study, the MOCART scores at 2 and 4 months postoperatively in the OAT group were 80 ± 7.07 and 75 ± 4.08, respectively, which are similar to the results reported by Keszég et al. [8].

Although OAT has shown excellent clinical results and histological outcomes, its disadvantages are also well known. Because of donor site morbidity, OAT is typically limited to lesions of less than 2 cm^2^. There are many challenges when using OAT with mosaicplasty for large osteochondral defects. For example, the filling rate cannot be 100% when more than two osteochondral plugs are used to repair the defect. Thus, there is inadequate healing of the osteochondral plugs with the surrounding bone and cartilage when the interface of the osteochondral plugs is not flush with the interface of the surrounding articular cartilage, and there is very often a mismatch between the radius of the curvature of the osteochondral plug and the surface of the donor area [30]. These disadvantages have limited the application of OAT in clinical practice.

In comparison with OAT, ADTT shows some advantages such as more donor sources of autologous cancellous bone and cartilage chips. Cancellous bone can be derived from the ilium and proximal tibia. Cartilage chips can be derived from the non-weightbearing zones of the knee joint, such as the intercondylar fossa, or even from other joints or rib cartilage. Furthermore, bone and cartilage can be taken from osteochondral fragments in the joint cavity. Therefore, ADTT can be applied to treat larger osteochondral defects than OAT. Furthermore, OAT does not allow for compression of the cancellous bone of the graft, resulting in poor intercancellous bone healing and sometimes resulting in a clear demarcation between transplanted and adjacent cancellous bone [39]. In contrast, the cancellous bone in ADTT is implanted under pressure and heals well with the adjacent cancellous bone, as confirmed in the present study.

ADTT is an extension of the use of ACC to treat osteochondral lesions. ACC shares similar indications to other cartilage restoration procedures such as ACI and MACI. The clinical indications of ACC include symptomatic tibiofemoral or patellofemoral cartilage defects, generally in patients younger than 55 years [43]. MACI was approved by the FDA in 2017 in the USA and is currently the only FDA-approved cell-based cartilage treatment option [30]. Some animal studies have demonstrated that ACC achieves results that are superior to the microfracture and similar to ACI [27]. A comparative study of the microfracture versus ACC embedded in fibrin glue in Göttingen minipigs concluded that the ACC transplant resulted in better quality cartilage repair tissue at 6 months postoperatively [44]. A study compared the in vitro results of treatment with allogeneic minced cartilage and allogeneic chondrocytes in a rabbit model [45]. Osteochondral defects at the trochlear groove were created in 56 rabbits, divided into the defect group, minced cartilage group (defect filled with allogeneic minced cartilage), ACI group (defect filled with isolated allogeneic chondrocytes embedded in atelocollagen gel), and atelocollagen with periosteal flap group (defect filled with atelocollagen gel) [45]. The authors concluded that implanting minced cartilage embedded in atelocollagen gel as a single-step procedure has similar outcomes to ACI but is cheaper and more convenient than ACI [45]. Therefore, the transplantation of ACC may be preferable to ACI in the treatment of cartilage defects, especially in countries where ACI/MACI is not available.

Although there is limited clinical evidence on autologous minced cartilage procedures and ADTT, the available data support the safety and satisfactory clinical outcomes of these procedures. Massen et al. [46] analyzed 27 consecutive patients with chondral or osteochondral lesions of the knee (mean cartilage defect 3.1 ± 1.6 cm^2^) treated using a single-step autologous minced cartilage procedure. The pain score significantly decreased from 7.2 ± 1.9 preoperatively to 1.8 ± 1.6 at 2 years postoperatively, while the mean knee function score improved from 7.2 ± 2.0 preoperatively to 2.1 ± 2.3 at 2 years postoperatively [46]. The mean MOCART score was 40.6 ± 21.1 at 6 months postoperatively [46]. Christensen et al. [31] reported the clinical outcomes of eight patients (mean age 32 ± 7.5 years) with OCD who received ADTT. The MOCART score for cartilage tissue repair improved from 22.5 preoperatively to 52.5 at 1 year postoperatively [31]. CT demonstrated very good subchondral bone healing, with bone filling of > 80% in all eight patients [31]. There were also improvements in all clinical scores (International Knee Documentation Committee score, Tegner score, and Knee Injury and Osteoarthritis Outcome Scores) [31]. The authors concluded that treatment of OCD with ADTT results in very good subchondral bone restoration and good cartilage repair [31]. More high-quality clinical and animal model studies are required to investigate the value of ADTT for the treatment of osteochondral defects.

The present study used a porcine model of osteochondral defects because the pig is a reliable model for studying cartilage repair. The pig joint size, weight requirements, and cartilage thickness are close to humans than dogs and smaller animal models. In addition, minipigs have a similar bone apposition rate and trabecular thickness to human [47]. A previous study showed that a 6.3-mm-diameter OCD lesion does not spontaneously heal in the minipig, confirming the applicability of this pig breed to articular cartilage research [48]. The empty control group in the present study also demonstrated a lack of self-healing ability for the 8-mm-diameter and 8-mm-deep osteochondral defects. However, the disadvantage of pigs is that they cannot be rested after surgery, and postoperative weightbearing activities of the knee may result in the detachment of the gel-fixed minced cartilage, leading to poor tissue healing.

Not all of the defects in the present study were well-healed. At 2 months postoperatively, one knee in the ADTT group had a circular-like depression of approximately 2 mm in diameter and 2 mm in depth in the central part of the defect; the ICRS score of this defect was 3, Grade IV, which indicates severely abnormal healing. Many variables determine the quality of the repair tissue after ADTT. Previous in vitro studies have demonstrated that juvenile cartilage has a greater proliferative ability than adult cartilage [49]. The use of adult pigs in the present study (mean age, 19.38 ± 1.06 months; range 18.0–21.5 months) may be one reason for the poor quality of the repair tissue. The degree of cartilage fragmentation is another important factor affecting the quality of the repair tissue. With both autologous adult and allogeneic juvenile chondral fragments, the current standard is to use 1–2 mm^3^ cartilage pieces [50]. However, the size of the chipped chondral fragments used in ADTT in the clinical study by Christensen et al. was 0.25–0.5 mm^2^ [31]. The smaller the cartilage fragments, the better the outward chondrocyte migration and the matrix synthesis, resulting in better repair tissue [51]. However, it is difficult in surgical practice to cut the cartilage into uniform particles of less than 0.5 mm^3^. Additionally, the postoperative weightbearing conditions without restrictions may also contributed to poor healing in the pigs.

The present study has some limitations. The sample size was small and the follow-up was short. The postoperative follow-up period is usually between 3 and 24 months when using the pig as a cartilage defect model to investigate cartilage or osteochondral repair [47]. Additionally, the osteochondral defects were acute rather than chronic, and there was no biomechanical testing of the repair tissue. Furthermore, as external brace devices cannot be used for pigs, there were no limitations on the range of motion and weightbearing during postoperative rehabilitation.

## Conclusions

We found that both ADTT and OAT are effective treatments for osteochondral defects in weightbearing areas in the Dian-nan small-ear pig model. Furthermore, ADTT achieved comparable results to OAT regarding the gross, radiographic, and histological evaluations of the repair tissue. The present findings suggest that ADTT can be used as an alternative procedure to OAT for treating osteochondral defects.

## Supplementary Information

**Additional file 1: Table S1.** ICRS macroscopic evaluation of cartilage repair. **Table S2.** MOCART scoring system of cartilage repair tissue based on MRI. **Table S3.** O'Driscoll histological score.

## Data Availability

The datasets used and analyzed during this study are available from the corresponding author on reasonable request, taking into account any confidentiality.

## Abbreviations

OAT: Osteochondral autograft transplantation
ADTT: Autologous dual-tissue transplantation
ICRS: International Cartilage Repair Society
MOCART: Magnetic resonance observation of cartilage repair tissue
CT: Computed tomography
OCD: Osteochondritis dissecans
ACI: Autologous chondrocyte implantation
MACI: Matrix-induced autologous chondrocyte implantation
ACC: Autologous cartilage chips
MRI: Magnetic resonance imaging

## References

[1] DiBartola AC, Wright BM, Magnussen RA, Flanigan DC (2016). Clinical outcomes after autologous chondrocyte implantation in adolescents' knees: a systematic review. Arthroscopy.

[2] Perera JR, Gikas PD, Bentley G (2012). The present state of treatments for articular cartilage defects in the knee. Ann R Coll Surg Engl.

[3] Habelt S, Hasler CC, Steinbruck K, Majewski M (2011). Sport injuries in adolescents. Orthop Rev (Pavia).

[4] Roemer FW, Kwoh CK, Hannon MJ, Green SM, Jakicic JM, Boudreau R (2012). Risk factors for magnetic resonance imaging-detected patellofemoral and tibiofemoral cartilage loss during a six-month period: the joints on glucosamine study. Arthritis Rheum.

[5] Guermazi A, Hayashi D, Roemer FW, Niu J, Quinn EK, Crema MD (2017). Brief report: partial- and full-thickness focal cartilage defects contribute equally to development of new cartilage damage in knee osteoarthritis: the multicenter osteoarthritis study. Arthritis Rheumatol.

[6] Brown TD, Johnston RC, Saltzman CL, Marsh JL, Buckwalter JA (2006). Posttraumatic osteoarthritis: a first estimate of incidence, prevalence, and burden of disease. J Orthop Trauma.

[7] Christensen BB (2016). Autologous tissue transplantations for osteochondral repair. Dan Med J.

[8] Keszég M, Hangody L, Egyed Z, Tóth G, Pánics G (2022). Long-term (10–25 years) outcomes of knee osteochondral autologous transplantation in soccer players. J Cartil Jt Preserv.

[9] Robinson A, Lindsay A, Vidal A, Frank RM (2020). Osteochondral autograft transfer (OATS). Oper Tech Sports Med.

[10] Solheim E, Hegna J, Oyen J, Harlem T, Strand T (2013). Results at 10 to 14 years after osteochondral autografting (mosaicplasty) in articular cartilage defects in the knee. Knee.

[11] Gudas R, Gudaite A, Pocius A, Gudiene A, Cekanauskas E, Monastyreckiene E (2012). Ten-year follow-up of a prospective, randomized clinical study of mosaic osteochondral autologous transplantation versus microfracture for the treatment of osteochondral defects in the knee joint of athletes. Am J Sports Med.

[12] Steadman JR, Briggs KK, Rodrigo JJ, Kocher MS, Gill TJ, Rodkey WG (2003). Outcomes of microfracture for traumatic chondral defects of the knee: average 11-year follow-up. Arthroscopy.

[13] Rikken QGH, Dahmen J, Reilingh ML, van Bergen CJA, Stufkens SAS, Kerkhoffs G (2021). Outcomes of bone marrow stimulation for secondary osteochondral lesions of the talus equal outcomes for primary lesions. Cartilage.

[14] Pareek A, Reardon PJ, Macalena JA, Levy BA, Stuart MJ, Williams RJ (2016). Osteochondral autograft transfer versus microfracture in the knee: a meta-analysis of prospective comparative studies at midterm. Arthroscopy.

[15] Knutsen G, Drogset JO, Engebretsen L, Grontvedt T, Isaksen V, Ludvigsen TC (2007). A randomized trial comparing autologous chondrocyte implantation with microfracture. Findings at five years. J Bone Jt Surg Am.

[16] Peterson L, Minas T, Brittberg M, Nilsson A, Sjogren-Jansson E, Lindahl A (2000). Two- to 9-year outcome after autologous chondrocyte transplantation of the knee. Clin Orthop Relat Res.

[17] Gilat R, Haunschild ED, Huddleston H, Parvaresh KC, Chahla J, Yanke AB (2021). Osteochondral allograft transplantation of the knee in adolescent patients and the effect of physeal closure. Arthroscopy.

[18] Gwosdz J, Rosinski A, Chakrabarti M, Woodall BM, Elena N, McGahan PJ (2019). Osteochondral allograft transplantation of the medial femoral condyle with orthobiologic augmentation and graft-recipient microfracture preparation. Arthrosc Tech.

[19] Wang T, Bugbee WD (2022). Osteochondral allograft transplantation in the football player (knee and ankle). J Cartil Jt Preserv.

[20] Pereira GF, Steele JR, Fletcher AN, Clement RD, Arasa MA, Adams SB (2021). Fresh osteochondral allograft transplantation for osteochondral lesions of the talus: a systematic review. J Foot Ankle Surg.

[21] Ao Y, Li Z, You Q, Zhang C, Yang L, Duan X (2019). The use of particulated juvenile allograft cartilage for the repair of porcine articular cartilage defects. Am J Sports Med.

[22] Zhang C, Ao Y, Cao J, Yang L, Duan X (2020). Donor cell fate in particulated juvenile allograft cartilage for the repair of articular cartilage defects. Am J Sports Med.

[23] Dunkin BS, Lattermann C (2013). New and emerging techniques in cartilage repair: MACI. Oper Tech Sports Med.

[24] Hevesi M, Krych AJ, Saris DBF (2019). Treatment of cartilage defects with the matrix-induced autologous chondrocyte implantation cookie cutter technique. Arthrosc Tech.

[25] Runer A, Salzmann GM (2022). Moving towards single stage cartilage repair—Is there evidence for the minced cartilage procedure?. J Cartil Joint Preserv.

[26] Wodzig MHH, Peters MJM, Emanuel KS, Van Hugten PPW, Wijnen W, Jutten LM (2022). Minced autologous chondral fragments with fibrin glue as a simple promising one-step cartilage repair procedure: a clinical and MRI study at 12-month follow-up. Cartilage.

[27] Salzmann GM, Ossendorff R, Gilat R, Cole BJ (2021). Autologous minced cartilage implantation for treatment of chondral and osteochondral lesions in the knee joint: an overview. Cartilage.

[28] Wei W, Dai H (2021). Articular cartilage and osteochondral tissue engineering techniques: recent advances and challenges. Bioact Mater.

[29] Lynch TS, Patel RM, Benedick A, Amin NH, Jones MH, Miniaci A (2015). Systematic review of autogenous osteochondral transplant outcomes. Arthroscopy.

[30] Krych AJ, Saris DBF, Stuart MJ, Hacken B (2020). Cartilage injury in the knee: assessment and treatment options. J Am Acad Orthop Surg.

[31] Christensen BB, Foldager CB, Jensen J, Lind M (2015). Autologous dual-tissue transplantation for osteochondral repair: early clinical and radiological results. Cartilage.

[32] van den Borne MPJ, Raijmakers NJH, Vanlauwe J, Victor J, de Jong SN, Bellemans J (2007). International Cartilage Repair Society (ICRS) and Oswestry macroscopic cartilage evaluation scores validated for use in autologous chondrocyte implantation (ACI) and microfracture. Osteoarthr Cartil.

[33] Marlovits S, Singer P, Zeller P, Mandl I, Haller J, Trattnig S (2006). Magnetic resonance observation of cartilage repair tissue (MOCART) for the evaluation of autologous chondrocyte transplantation: determination of interobserver variability and correlation to clinical outcome after 2 years. Eur J Radiol.

[34] Marlovits S, Striessnig G, Resinger CT, Aldrian SM, Vecsei V, Imhof H (2004). Definition of pertinent parameters for the evaluation of articular cartilage repair tissue with high-resolution magnetic resonance imaging. Eur J Radiol.

[35] Quirbach S, Trattnig S, Marlovits S, Zimmermann V, Domayer S, Dorotka R (2009). Initial results of in vivo high-resolution morphological and biochemical cartilage imaging of patients after matrix-associated autologous chondrocyte transplantation (MACT) of the ankle. Skelet Radiol.

[36] Moran ME, Kim HK, Salter RB (1992). Biological resurfacing of full-thickness defects in patellar articular cartilage of the rabbit. Investigation of autogenous periosteal grafts subjected to continuous passive motion. J Bone Jt Surg Br.

[37] Hangody L, Karpati Z (1994). New possibilities in the management of severe circumscribed cartilage damage in the knee. Magy Traumatol Ortop Kezseb Plasztikai Seb.

[38] de l’Escalopier N, Amouyel T, Mainard D, Lopes R, Cordier G, Baudrier N (2021). Long-term outcome for repair of osteochondral lesions of the talus by osteochondral autograft: a series of 56 Mosaicplasties(R). Orthop Traumatol Surg Res.

[39] Tibesku CO, Szuwart T, Kleffner TO, Schlegel PM, Jahn UR, Van Aken H (2004). Hyaline cartilage degenerates after autologous osteochondral transplantation. J Orthop Res.

[40] Kleemann RU, Schell H, Thompson M, Epari DR, Duda GN, Weiler A (2007). Mechanical behavior of articular cartilage after osteochondral autograft transfer in an ovine model. Am J Sports Med.

[41] Harman BD, Weeden SH, Lichota DK, Brindley GW (2006). Osteochondral autograft transplantation in the porcine knee. Am J Sports Med.

[42] Baumbach K, Petersen J-P, Ueblacker P, Schröder J, Göpfert C, Stork A (2008). The fate of osteochondral grafts after autologous osteochondral transplantation: a one-year follow-up study in a minipig model. Arch Orthop Trauma Surg.

[43] Gilat R, Haunschild ED, Knapik DM, Cole BJ (2020). Single-stage minced autologous cartilage restoration procedures. Oper Tech Sports Med.

[44] Christensen BB, Olesen ML, Lind M, Foldager CB (2017). Autologous cartilage chip transplantation improves repair tissue composition compared with marrow stimulation. Am J Sports Med.

[45] Matsushita R, Nakasa T, Ishikawa M, Tsuyuguchi Y, Matsubara N, Miyaki S, Adachi N (2019). Repair of an osteochondral defect with minced cartilage embedded in atelocollagen gel: a rabbit model. Am J Sports Med.

[46] Massen FK, Inauen CR, Harder LP, Runer A, Preiss S, Salzmann GM (2019). One-step autologous minced cartilage procedure for the treatment of knee joint chondral and osteochondral lesions: a series of 27 patients with 2-year follow-up. Orthop J Sports Med.

[47] Meng X, Ziadlou R, Grad S, Alini M, Wen C, Lai Y (2020). Animal models of osteochondral defect for testing biomaterials. Biochem Res Int.

[48] Gotterbarm T, Breusch SJ, Schneider U, Jung M (2008). The minipig model for experimental chondral and osteochondral defect repair in tissue engineering: retrospective analysis of 180 defects. Lab Anim.

[49] Zhang C, Zhao X, Ao Y, Cao J, Yang L, Duan X (2021). Proliferation ability of particulated juvenile allograft cartilage. J Orthop Surg Res.

[50] Cole BJ, Farr J, Winalski CS, Hosea T, Richmond J, Mandelbaum B (2011). Outcomes after a single-stage procedure for cell-based cartilage repair: a prospective clinical safety trial with 2-year follow-up. Am J Sports Med.

[51] Bonasia DE, Marmotti A, Mattia S, Cosentino A, Spolaore S, Governale G (2015). The degree of chondral fragmentation affects extracellular matrix production in cartilage autograft implantation: an in vitro study. Arthrosc J Arthrosc Relat Surg.

