# Supplementary material for: Dual-tissue transplantation versus osteochondral autograft transplantation in the treatment of osteochondral defects: a porcine model study
Source: J Orthop Surg Res. 2023 Jul 5;18:481. doi: 10.1186/s13018-023-03964-6 (PMC10321008; doi:10.1186/s13018-023-03964-6)
Supplement: Supplementary file 1 — Additional file 1: Table S1. ICRS macroscopic evaluation of cartilage repair. Table S2. MOCART scoring system of cartilage repair tissue based on MRI. Table S3. O'Driscoll histological score. [file 13018_2023_3964_MOESM1_ESM.docx]

Additional file 1: Table S1. ICRS macroscopic evaluation of cartilage repair

| Cartilage repair assessment ICRS | Points |
| --- | --- |
| Degree of defect repair  In level with surrounding cartilage  75% repair of defect depth  50% repair of defect depth  25% repair of defect depth  0% repair of defect depth | 4  3  2  1  0 |
| Integration to border zone  Complete integration with surrounding cartilage  Demarcating border < 1 mm  3/4th of graft integrated, 1/4th with a notable border > 1 mm width  1/2 of graft integrated with surrounding cartilage, 1/2 with a notable border > 1 mm  From no contact to 1/4th of graft integrated with surrounding cartilage | 4  3  2  1  0 |
| Macroscopic appearance  Intact smooth surface  Fibrillated surface  Small, scattered fissures or cracs  Several, small or few but large fissures  Total degeneration of grafted area | 4  3  2  1  0 |
| Overall repair assessment  Grade I: normal  Grade II: nearly normal  Grade III: abnormal  Grade IV: severely abnormal | 12  11-8  7-4  3-1 |

Additional file 1: Table S2. MOCART scoring system of cartilage repair tissue based on MRI

| Variables | Points |
| --- | --- |
| Degree of defect repair and filling of the defect  Complete  Hypertrophy  Incomplete  >50% of the adjacent cartilage  <50% of the adjacent cartilage  Subchondral bone exposed | 20  15  10  5  0 |
| Integration to border zone  Complete  Incomplete  Demarcating border visible (slit like)  Defect visible <50% of the length  Defect visible >50% of the length | 15  10  5  0 |
| Surface of the repair tissue  Surface intact  Surface damaged <50% of depth  Surface damaged >50% of depth | 10  5  0 |
| Structure of the repair tissue  Homogeneous  Inhomogeneous | 5  0 |
| Signal intensity of the repair tissue  Normal (identical to adjacent cartilage)  Nearly normal (slight areas of signal alteration)  Abnormal (large areas of signal alteration) | 30  15  0 |
| Subchondral lamina  Intact  Not intact | 5  0 |
| Subchondral bone  Intact  Not intact | 5  0 |
| Adhesions  No  Yes | 5  0 |
| Effusion  No  Yes | 5  0 |

Additional file 1: Table S3．O'Driscoll histological score

| Variables | Points |
| --- | --- |
| 1. Nature of the predominant tissue   1.Cellular morphology  Hyaline articular cartilage  Incompletely differentiated mesenchyme  Fibrous tissue or bone | 4  2  0 |
| 2.Safranin-O staining of matrix  Normal or nearly normal  Moderate  Slight  None | 3  2  1  0 |
| 1. Structural characteristics   3. Surface regularity  Smooth and intact  Superficial horizontal lamination  Fissure 25 to 100 percent of the thickness  Severe disruption including fibrillation | 3  2  1  0 |
| 4. Structural integrity  Normal  Slight disruption including cysts  Severe disintegration | 2  1  0 |
| 5. Thickness  100 percent of normal adjacent cartilage  50-100 percent of normal cartilage  0-50 percent of normal cartilage | 2  2  0 |
| 6. Bonding to the adjacent cartilage  Bonded at both ends of graft  Bonded at one end, or partially at both ends  Not bonded | 2  1  0 |
| 1. Freedom from cellular changes of degeneration   7. Hypocellularity  Normal cellularity  Slight hypocellularity  Moderate hypocellularity  Severe hypocellularity | 3  2  1  0 |
| 8. Chondrocyte clustering  No clusters  <25 percent of cells  25-100 percent of cells | 2  1  0 |
| 9. Freedom from degenerative changes in adjacent cartilage  Normal cellularity, no clusters, normal staining  Normal cellularity, mild clusters, moderate staining  Mild or moderate hypocellularity, slight staining  Severe hypocellularity, poor or no staining | 3  2  1  0 |
